# Automating Stroke Data Extraction From Free-Text Radiology Reports Using Natural Language Processing: Instrument Validation Study

**DOI:** 10.2196/24381

**Published:** 2021-05-04

**Authors:** Amy Y X Yu, Zhongyu A Liu, Chloe Pou-Prom, Kaitlyn Lopes, Moira K Kapral, Richard I Aviv, Muhammad Mamdani

**Affiliations:** 1 Department of Medicine (Neurology) University of Toronto – Sunnybrook Health Sciences Centre Toronto, ON Canada; 2 Unity Health Toronto Toronto, ON Canada; 3 Department of Medicine (General Internal Medicine) University of Toronto – University Health Network Toronto, ON Canada; 4 Department of Radiology Division of Neuroradiology University of Ottawa Ottawa, ON Canada; 5 Department of Medicine Unity Health Toronto University of Toronto Toronto, ON Canada

**Keywords:** stroke, diagnostic imaging, data extraction, natural language processing, neurovascular, imaging, stroke surveillance, surveillance

## Abstract

**Background:**

Diagnostic neurovascular imaging data are important in stroke research, but obtaining these data typically requires laborious manual chart reviews.

**Objective:**

We aimed to determine the accuracy of a natural language processing (NLP) approach to extract information on the presence and location of vascular occlusions as well as other stroke-related attributes based on free-text reports.

**Methods:**

From the full reports of 1320 consecutive computed tomography (CT), CT angiography, and CT perfusion scans of the head and neck performed at a tertiary stroke center between October 2017 and January 2019, we manually extracted data on the presence of proximal large vessel occlusion (primary outcome), as well as distal vessel occlusion, ischemia, hemorrhage, Alberta stroke program early CT score (ASPECTS), and collateral status (secondary outcomes). Reports were randomly split into training (n=921) and validation (n=399) sets, and attributes were extracted using rule-based NLP. We reported the sensitivity, specificity, positive predictive value (PPV), negative predictive value (NPV), and the overall accuracy of the NLP approach relative to the manually extracted data.

**Results:**

The overall prevalence of large vessel occlusion was 12.2%. In the training sample, the NLP approach identified this attribute with an overall accuracy of 97.3% (95.5% sensitivity, 98.1% specificity, 84.1% PPV, and 99.4% NPV). In the validation set, the overall accuracy was 95.2% (90.0% sensitivity, 97.4% specificity, 76.3% PPV, and 98.5% NPV). The accuracy of identifying distal or basilar occlusion as well as hemorrhage was also high, but there were limitations in identifying cerebral ischemia, ASPECTS, and collateral status.

**Conclusions:**

NLP may improve the efficiency of large-scale imaging data collection for stroke surveillance and research.

## Introduction

Stroke is a leading cause of death and disability [1]. Neuroimaging study findings inform treatment and prognosis. For example, recent clinical trials have demonstrated the efficacy of endovascular thrombectomy, a mechanical clot-retrieval procedure, in improving functional outcomes in patients with acute ischemic stroke and proximal large vessel occlusion [2-5]. Data on efficacy of this procedure in patients with distal or smaller vessel occlusion are currently lacking. Although large health administrative databases have information on whether a stroke was ischemic or hemorrhagic, detailed neuroimaging findings are usually found in narrative diagnostic imaging reports and obtained through resource-intensive manual chart abstractions [6,7].

The lack of population-based neuroimaging data limits the ability to characterize the prevalence of large vessel occlusion. A recent meta-analysis of cohort studies of patients with ischemic stroke found that the prevalence of large vessel occlusion ranged widely, from 13% to 52% [8], suggesting that smaller cohort studies can be vulnerable to selection bias. Therefore, automating the extraction of information on vessel occlusion from diagnostic imaging reports is needed for population-based disease surveillance and clinical research.

Natural language processing (NLP) can convert large amounts of free-text data into structured data and has been used to extract information on stroke type and location from diagnostic imaging reports [9-11]. However, its ability to characterize vascular occlusions is not well understood. We aimed to determine the accuracy of an NLP tool [12] in identifying the presence and location of vascular occlusions and other stroke-related attributes from neuroimaging reports of computed tomography (CT), CT angiography (CTA), and CT perfusion (CTP) scans. We hypothesized that an NLP tool can identify large vessel occlusion with high accuracy.

## Methods

### Manual Chart Abstraction

We obtained full free-text reports of 1320 consecutive stroke protocol imaging studies comprising CT, CTA, and CTP imaging of the head and neck performed between October 2017 and January 2019 at a university-affiliated comprehensive stroke center that provides consultation for endovascular thrombectomy to a catchment area of 2.5 million people. A stroke specialist and a trained research assistant manually extracted stroke-related attributes from the reports. The primary outcome was the presence of large vessel occlusion defined as occlusion in the M1 segment of the middle cerebral artery (MCA-M1) or A1 segment of the anterior cerebral artery (ACA-A1) with or without involvement of the carotid terminus because occlusion at these sites is treatable with endovascular thrombectomy. We chose this as the primary outcome because patients with this type of occlusion can be treated with endovascular thrombectomy. Isolated intracranial internal carotid artery occlusion was not categorized as large vessel occlusion in this study because the effectiveness of endovascular thrombectomy has not been shown in this population [13].

Secondary outcomes included (1) the presence of cerebral ischemia, (2) Alberta stroke program early CT score (ASPECTS) [14], (3) the presence of any intracranial hemorrhage, (4) distal anterior circulation occlusion defined as occlusion in the middle or anterior cerebral arteries in the M2 or A2 segments or beyond, (5) basilar occlusion, and (6) qualitative measure of collateral status (ie, good, intermediate, or poor). The manually extracted data were considered the reference standard. Duplicate chart abstraction on 200 charts showed that the inter-rater reliability was >96% for all attributes except for the presence of cerebral ischemia for which it was 80%. We randomly split the reports into training (n=921) and validation (n 399) sets.

### CHARTextract NLP Tool

NLP rule sets for stroke attribute extraction from free-text diagnostic imaging reports were created using CHARTextract version 0.3.2, freely available online [12]. CHARTextract is a rule-based information extraction tool that relies on regular expressions and works at the sentence level to identify word patterns. We opted to use a rule-based approach due to the small sample size and the availability of domain experts to develop and refine the rules.

**Figure 1:**
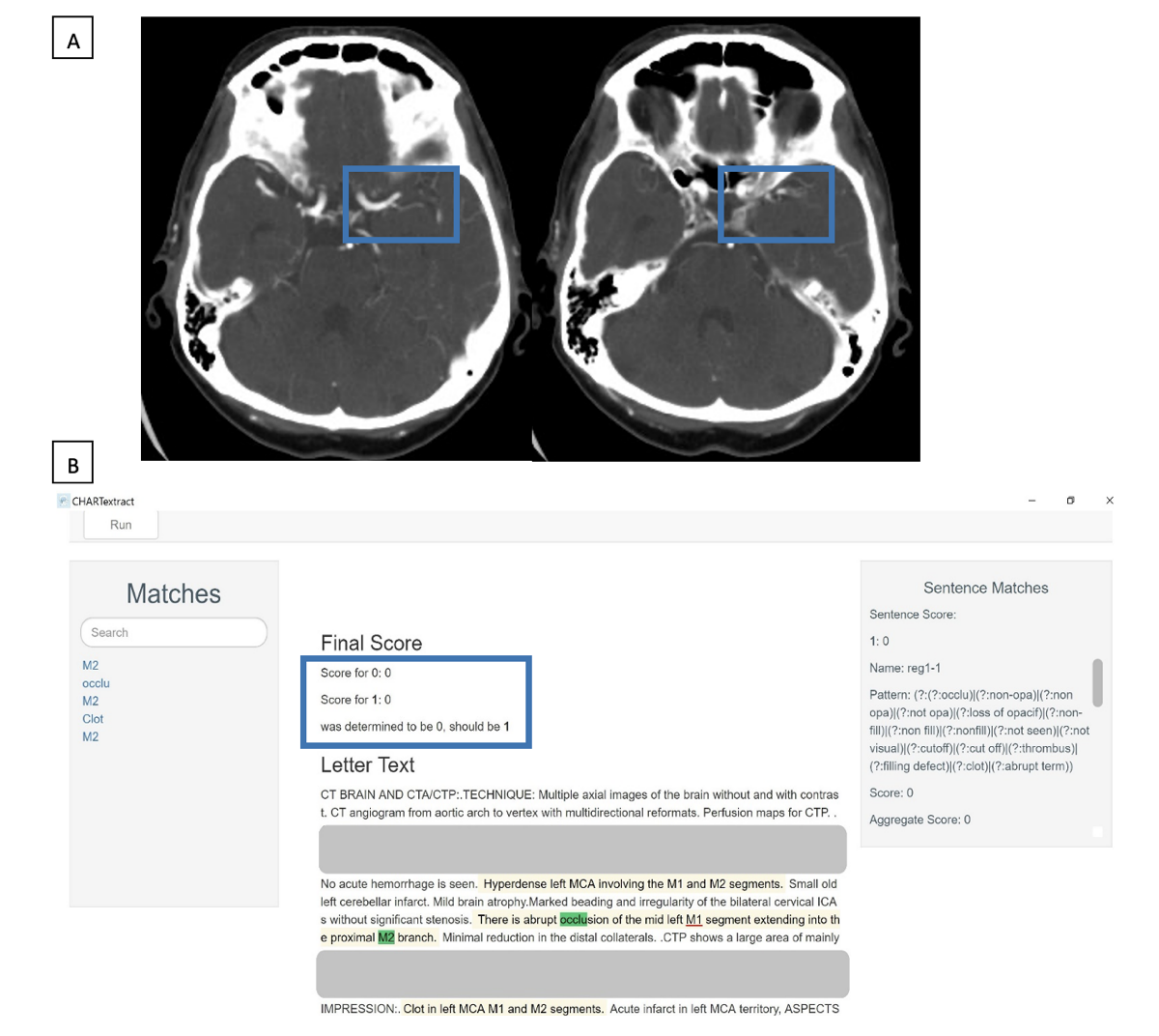
Example 1 of a discrepancy between the chart abstractor and CHARTextract tool output. (A) Computed tomography angiography scan showing loss of opacification in the left middle cerebral artery, involving the left M1 segment and extending into the M2 segment. (B) CHARTextract tool output: the chart abstractor labeled that large vessel occlusion was present, but the CHARTextract tool determined this attribute to be absent. The rules were revised to reflect that occlusion involving the “M1 segment” should be considered a large vessel occlusion even if the terms “MCA” or “middle cerebral artery” were absent.

We created information extraction pipelines by using an iterative process where each rule was assigned a weight by the end-user in the training set. For example, if a report contains the text “presence of middle cerebral artery occlusion…,” the system’s estimate of the probability of a large vessel occlusion increases; however, if a report contains the text “no evidence of…,” it will lower the system’s estimate of the probability. As shown in Figure 1, the tool displays the discrepancies between the chart abstractor label and the tool’s prediction, thus allowing for rapid iterative refinement of the rules by the end user. Rules were developed for each attribute through an iterative process by the end-user (ZL, AY, and CP) by using the training set that was validated in the validation set. For the presence of large vessel occlusion (our primary outcome), we also recorded whether the discrepancy between the chart abstractor and the NLP tool was due to abstractor or tool error. The rules thus developed are shown in Multimedia Appendix 1.

### Statistical Methods

The stroke-related attributes identified by the NLP tool, CHARTextract version 0.3.2, were compared to the reference standard. The sensitivity, specificity, positive predictive value (PPV), and negative predictive value (NPV) were calculated using this tool.

### Ethics Approval

The study was approved by the Sunnybrook Health Sciences Centre and Unity Health Toronto Research Ethics Boards with a waiver of individual patient consent prior to data collection.

## Results

Among the 1320 consecutive diagnostic imaging reports manually reviewed, chart abstractors identified 184 large vessel occlusions (MCA-M1, n=157; ACA-A1, n=27) in 161 (12.2%) reports. Distal anterior circulation occlusion was reported in 188 (14.2%) scans, basilar artery occlusion in 26 (2.0%) scans, established ischemia in 391 (29.6%) scans, and intracranial hemorrhage in 139 (10.5%) scans. ASPECTS was reported only in 384 (29.1%) reports (ASPECTS <5, n=40; ASPECTS ≥5, n=344), and collateral status was described in 216 (16.4%) reports (good, n=141; intermediate, n=26; poor, n=49).

**Figure 2:**
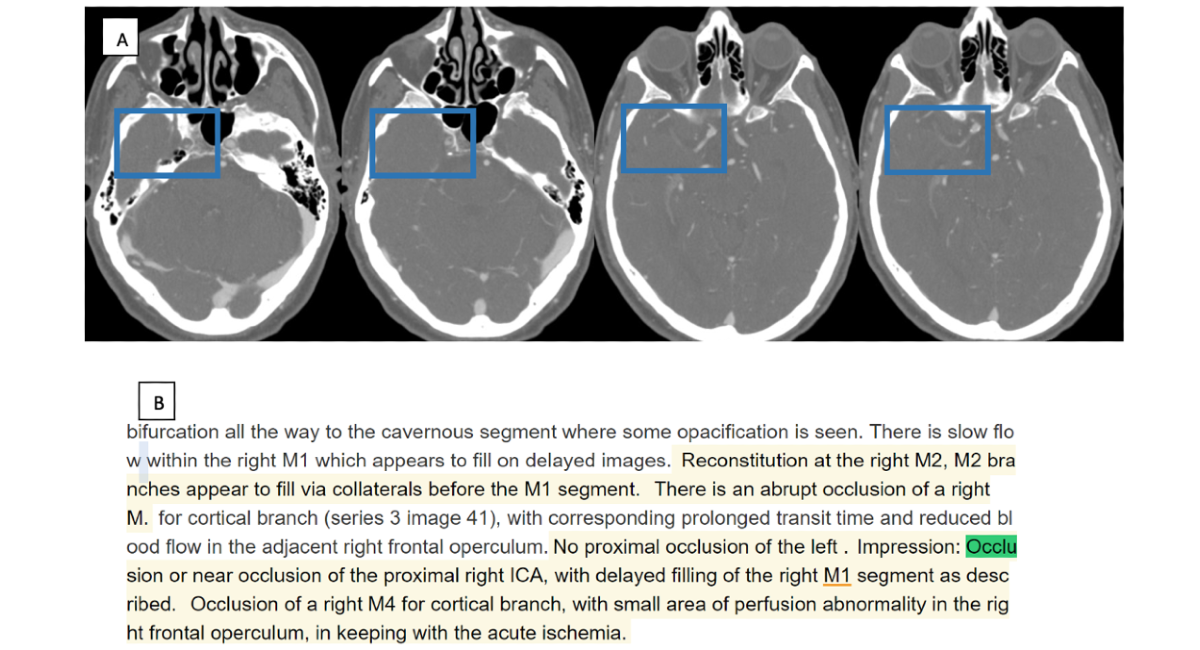
Example 2 of a discrepancy between the chart abstractor and CHARTextract tool output. (A) Computed tomography angiography scan showing near-occlusion of the cavernous internal carotid artery with reconstitution of the middle cerebral artery. (B) CHARTextract output: the abstractor labeled that large vessel occlusion was absent, but the CHARTextract tool determined this attribute to be present because the words “occlusion” and “M1 segment” were detected in the same sentence.

**Figure 3:**
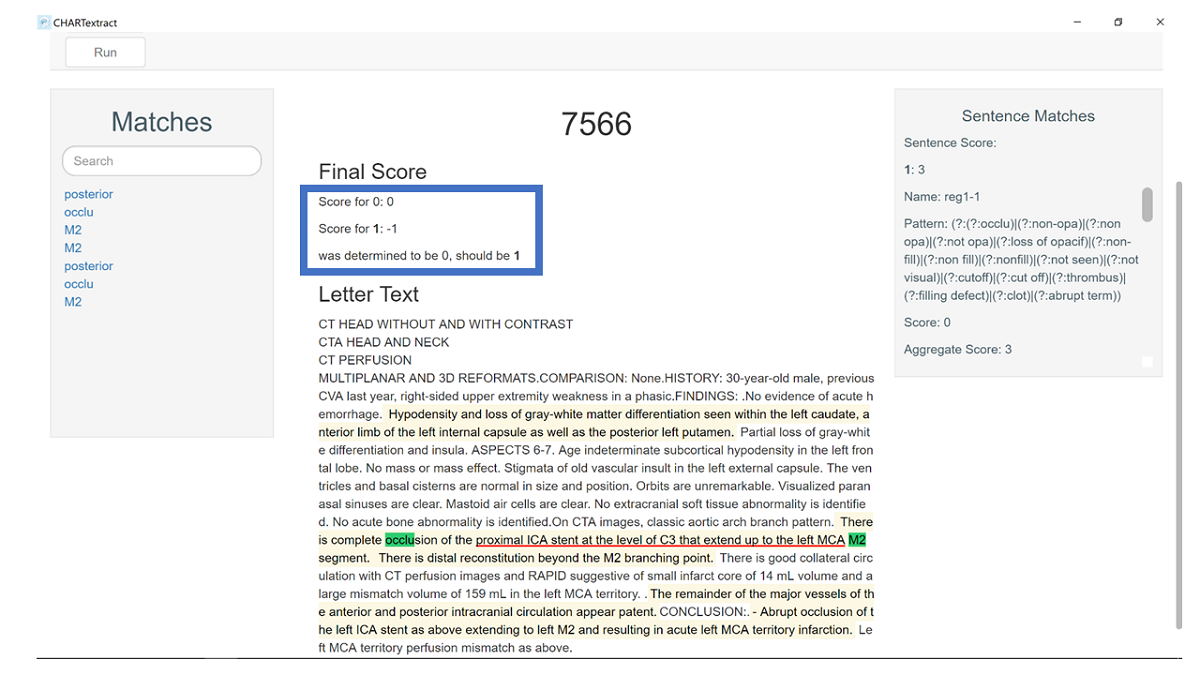
Example 3 of a discrepancy between the chart abstractor and CHARTextract tool output. The abstractor labeled that large vessel occlusion was present because the abstractor was able to interpret that an occlusion from the internal carotid artery and extending to the M2 segment of the middle cerebral artery involves the M1 segment, but the CHARTextract tool determined this attribute to be absent because the tool detects key words without knowledge of vascular anatomy.

Compared to the reference standard, the NLP tool identified large vessel occlusion with an overall accuracy of 97.3% (95.5% sensitivity, 98.1% specificity, 84.1% PPV, and 99.4% NPV). Despite an iterative process to refine rules, some scenarios remained challenging to translate into rules. Figure 2 illustrates an example wherein the CHARTextract tool determined large vessel occlusion to be present because the words “occlusion” and “M1 segment” were detected in the same sentence, but the report indicated that the occlusion was in the cavernous portion of the internal carotid artery with reconstitution of blood flow in the M1 segment. In another example illustrated in Figure 3, the CHARTextract tool determined that large vessel occlusion was absent because the report indicated the presence of an occlusion extending from the internal carotid artery to the M2 segment. Here, the tool only detected “internal carotid artery” and “M2” as keywords and could not interpret the vascular anatomy described in the report. Nevertheless, in the validation set, the overall accuracy for large vessel occlusion was still high at 95.2% (90.0% sensitivity, 97.4% specificity, 76.3% PPV, and 98.5% NPV). We also found that two of the 25 discrepancies between the abstractors and the NLP tool were due to chart abstractor error.

The accuracy of the CHARTextract tool for the other stroke attributes is presented in Table 1. The tool identified these other attributes with moderately high accuracy except for presence of established ischemia, which had a lower sensitivity and PPV of 82.2% and 80.5%, respectively, in the derivation cohort and 80.8% and 64.1%, respectively, in the validation cohort. The other exception was basilar occlusion, which was only present in 2.0% (26/1320) of the reports. Although the sensitivity and PPV for basilar occlusion were 100% and 95.0%, respectively, in the derivation cohort, the corresponding values were lower in the validation cohort (ie, 71.4% and 41.7%)

**Table 1:** Accuracy of the natural language processing tool CHARTextract to identify stroke-related attributes in diagnostic imaging reports.

Cohort and stroke-related attribute		Attribute prevalence, n (%)	Sensitivity (%)	Specificity(%)	PPV^a^ (%)	NPV^b^ (%)	Overall accuracy (%)
**Derivation cohort (n=921)**							
	Anterior proximal occlusion	111 (12.1)	95.5	98.1	84.1	99.4	97.3
	Anterior distal occlusion	127 (13.8)	92.9	98.0	88.1	98.9	97.3
	Basilar occlusion	19 (2.1)	100	99.9	95.0	100	99.9
	Presence of established ischemia	287 (31.2)	82.2	91.7	80.5	91.9	88.3
	Presence of any hemorrhage	114 (12.4)	93.0	98.2	87.6	99.0	97.5
**Validation cohort (n=399)**							
	Anterior proximal occlusion	50 (12.5)	90.0	97.4	76.3	98.5	95.2
	Anterior distal occlusion	61 (15.3)	83.6	97.7	86.4	97.1	95.5
	Basilar occlusion	7 (1.8)	71.4	98.2	41.7	99.5	97.7
	Presence of established ischemia	104 (26.1)	80.8	85.1	64.1	92.5	83.2
	Presence of any hemorrhage	25 (6.3)	88.0	96.0	59.5	99.2	95.5

^a^PPV: positive predictive value.

^b^NPV: negative predictive value.

The metrics for ASPECTS and collateral status are shown separately because data were incomplete (Table 2). Importantly, we found that the NLP tool was able to identify the reports with missing data with high accuracy. For example, information on ASPECTS was absent in 71.8% (661/921) of the reports in the derivation cohort and 68.99% (275/399) for the validation cohort. The tool accurately identified that this attribute was missing with a sensitivity and PPV of 99.7% and 99.7%, respectively, in the derivation cohort and 99.3% and 98.6%, respectively, in the validation cohort.

**Table 2:** Accuracy of the natural language processing tool CHARTextract to identify Alberta stroke program early CT score (ASPECTS) and collateral vascular status based on diagnostic imaging reports.

Cohort and stroke-related attributes			Attribute prevalence, n (%)	Sensitivity (%)		Specificity (%)	PPV^a^ (%)	NPV^b^ (%)	Overall accuracy (%)	
**Derivation cohort (n=921)**										
	**ASPECTS**									98.8
		Not reported	661 (71.8)		99.7	99.2	99.7	99.2		
		<5	30 (3.3)		96.7	99.2	80.6	99.9		
		≥5	230 (25.0)		96.5	99.7	99.1	98.9		
	**Collateral status**									98.4
		Not reported	774 (84.0)		99.2	96.6	99.4	95.9		
		Poor	34 (3.7)		94.1	100	100	99.8		
		Intermediate	19 (2.1)		78.9	100	100	99.6		
		Good	94 (10.2)		96.8	98.8	90.1	99.6		
**Validation cohort (n=399)**										
	**ASPECTS**									98.5
		Not reported	275 (68.9)		99.3	96.8	98.6	98.4		
		<5	10 (2.5)		70.0	100	100.0	99.2		
		≥5	114 (28.6)		99.1	99.3	98.3	99.6		
	**Collateral status**									98.2
		Not reported	330 (82.7)		99.7	91.3	98.2	98.4		
		Poor	15 (3.8)		93.3	99.7	93.3	99.7		
		Intermediate	7 (1.8)		71.4	100	100	99.5		
		Good	47 (11.8)		93.6	100	100	99.2		

^a^PPV: positive predictive value.

^b^NPV: negative predictive value.

## Discussion

### Principal Findings

We showed that an NLP approach can automate data extraction from neuroimaging reports with moderately high accuracy, supporting its potential application for stroke surveillance, health system planning, and population-based clinical research. The PPV of CHARTextract to identify large vessel occlusion was 76.3%, meaning that of 100 reports identified to have a large vessel occlusion, there were 24 false-positive cases, but the sensitivity, specificity, and NPV were over 90%, indicating the prevalence of fewer false-negative cases. Thus, NLP may be a helpful screening tool for case finding purposed when using a large dataset.

Although we did not formally record the time required for data abstraction, the abstractors estimate an average review time of 5 minutes per chart, which adds to 110 hours of sustained attention to review a total of 1320 charts. On the other hand, once the rule sets have been developed, the NLP tool can extract the requested variables within seconds.

### Limitations

There are several limitations of NLP that are worth discussing. First, the NLP approach can only extract information from the radiologist’s reported interpretation of diagnostic images, and it is not designed to be directly used for imaging interpretation [4]. Although the tool was accurate in identifying which reports had missing data on ASPECTS and collateral status, information on these attributes was simply not obtainable without the direct assessment of the images. Second, each rule is applied at a sentence level so that the tool will not be able to capture attributes if keywords occur across different sentences. Third, the tool does not distinguish between homonyms in the English language. For instance, we experienced challenges with the word “ASPECT” used to describe the score and “aspect” used to describe a facet of the brain or a component of a blood vessel. Finally, the NLP approach is influenced by variations in reporting practices to describe imaging findings. This was most apparent in the evaluation of the presence of cerebral ischemia. The terms used to describe this attribute were less predictable and frequently contained ambiguous language such as “possible subtle hypodensity” or “cannot rule out early ischemia.” Interestingly, the cerebral ischemia attribute also had a lower inter-rater reliability between the chart abstractors compared to the other attributes evaluated. We noticed that the nonclinical research assistant, who has extensive experience with chart abstraction for stroke research, was more liberal in recording ischemia, whereas the stroke specialist was more selective in recording ischemia depending on the language used by the radiologist. In this situation, the application of NLP rule sets may improve the standardization of data collection. Finally, the current proof-of-concept study has a small sample size. External validation of our methods with a larger sample of radiology reports is needed to address the limitations arising from variation in reporting practices.

### Conclusions

NLP approaches can identify the presence of large vessel occlusion with high accuracy and have the potential to improve the efficiency of large-scale data collection from imaging reports. External validation of our approach is needed.

## Appendix

Multimedia Appendix 1 CHARTextract tool rules.

## Abbreviations

ACA-A1: A1 segment of the anterior cerebral artery
ASPECTS: Alberta stroke program early CT score
CT: computed tomography
CTA: computed tomography angiography
CTP: computed tomography perfusion
MCA-M1: M1 segment of the middle cerebral artery
NLP: natural language processing
NPV: negative predictive value
PPV: positive predictive value
