# Supplementary material for: Automating Stroke Data Extraction From Free-Text Radiology Reports Using Natural Language Processing: Instrument Validation Study
Source: JMIR Med Inform. 2021 May 4;9(5):e24381. doi: 10.2196/24381 (PMC8132979; doi:10.2196/24381)
Supplement: Multimedia Appendix 1 [file medinform_v9i5e24381_app1.pdf]

Supplemental Figure 1 Rules for anterior proximal occlusion

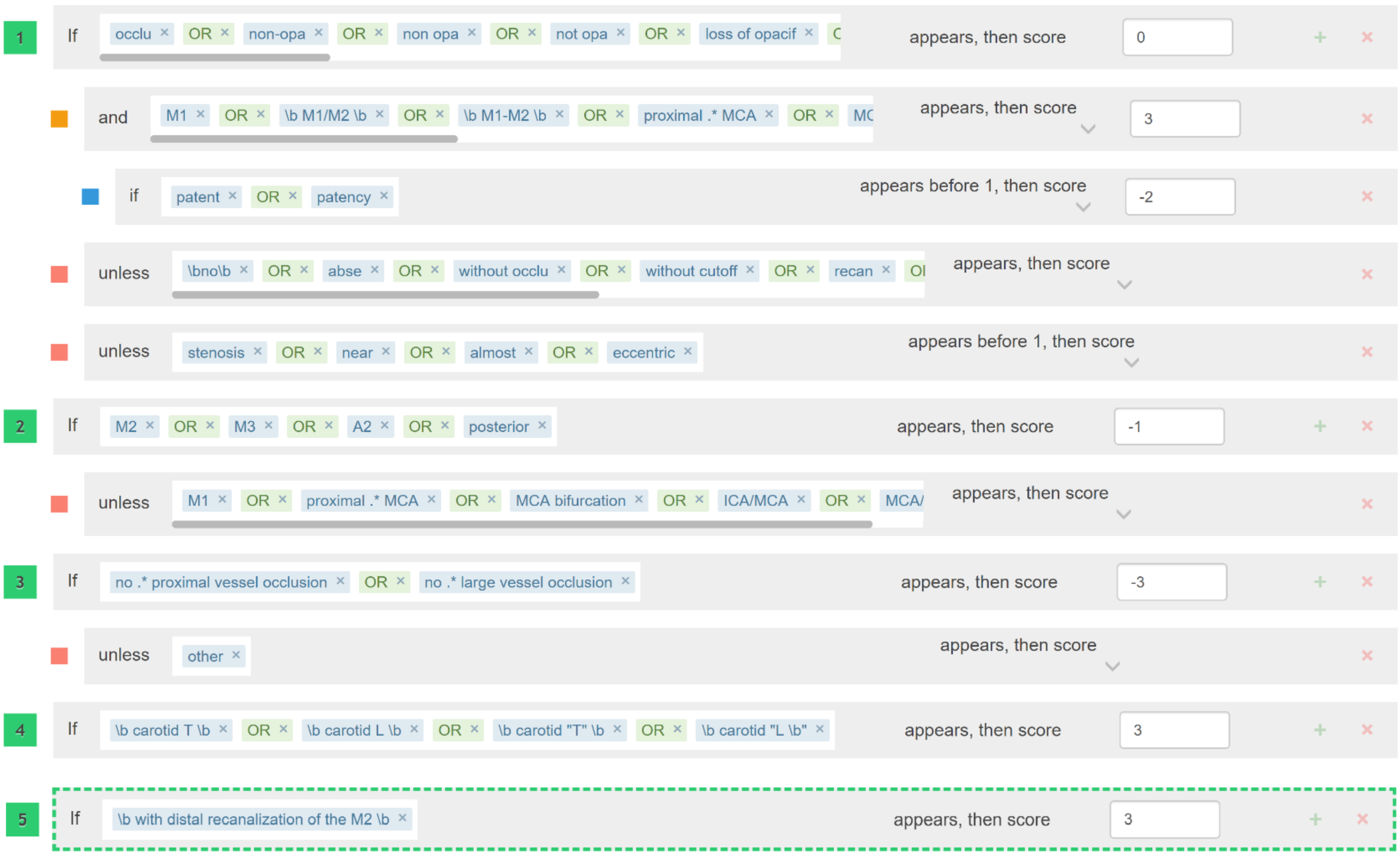

Rules for anterior proximal occlusion (text format):

"Name": "1",
"Dirty": false,
"Rules": [
{
"Primary": {
"Rule": [
"occlu",
"OR",
"non-opa",
"OR",
"non opa",
"OR",
"not opa",
"OR",
"loss of opacif",
"OR",
"non-fill",
"OR",
"non fill",
"OR",
"nonfill",
"OR",

```
        "not seen",
        "OR",
        "not visual",
        "OR",
        "cutoff",
        "OR",
        "cut off",
        "OR",
        "thrombus",
        "OR",
        "filling defect",
        "OR",
        "clot",
        "OR",
        "abrupt term"
    ],
    "Score": 0,
    "Selected": false,
    "type": "Primary",
    "u_id": "crqoyt"
},
"Secondary": {
    "Replace": [
        {
            "Rule": [
                "M1",
                "OR",
                "\\b M1/M2 \\b",
                "OR",
                "\\b M1-M2 \\b",
                "OR",
                "proximal .* MCA",
                "OR",
                "MCA bifurcation",
                "OR",
                "MCA .* bifurcation",
                "OR",
                "ICA/MCA",
                "OR",
                "ICA and MCA",
                "OR",
                "MCA/ICA",
                "OR",
                "A1"
            ],
            "Score": 3,
            "Modifier": "None",
            "Selected": false,
            "type": "Replace",
            "u_id": "yq2y5a"
        }
    ],
    "Ignore": [
        {
            "Rule": [
                "\\bno\\b",
                "OR",
                "abse",
```

```
          "OR",
          "without occlu",
          "OR",
          "without cutoff",
          "OR",
          "recan",
          "OR",
          "partial",
          "OR",
          "near tot",
          "OR",
          "non-occl",
          "OR",
          "nonoccl"
        ],
        "Score": 0,
        "Modifier": "None",
        "Selected": false,
        "type": "Ignore",
        "u_id": "gj53e"
      },
      {
        "Rule": [
          "stenosis",
          "OR",
          "near",
          "OR",
          "almost",
          "OR",
          "eccentric"
        ],
        "Score": 0,
        "Modifier": "b",
        "Selected": false,
        "type": "Ignore",
        "u_id": "zzol4o"
      }
    ],
    "Add": [
      {
        "Rule": [
          "patent",
          "OR",
          "patency"
        ],
        "Score": -2,
        "Modifier": "b",
        "Selected": false,
        "type": "Add",
        "u_id": "zt3j"
      }
    ]
  }
},
{
  "Primary": {
    "Rule": [
      "M2",
```

```
            "OR",
            "M3",
            "OR",
            "A2",
            "OR",
            "posterior"
         ],
         "Score": -1,
         "Selected": false,
         "type": "Primary",
         "u_id": "62wswd"
      },
      "Secondary": {
         "Replace": [],
         "Ignore": [
            {
               "Rule": [
                  "M1",
                  "OR",
                  "proximal .* MCA",
                  "OR",
                  "MCA bifurcation",
                  "OR",
                  "ICA/MCA",
                  "OR",
                  "MCA/ICA"
               ],
               "Score": 0,
               "Modifier": "None",
               "Selected": false,
               "type": "Ignore",
               "u_id": "2f2zqf"
            }
         ],
         "Add": []
      }
   },
   {
      "Primary": {
         "Rule": [
            "no .* proximal vessel occlusion",
            "OR",
            "no .* large vessel occlusion"
         ],
         "Score": -3,
         "Selected": false,
         "type": "Primary",
         "u_id": "7oemce"
      },
      "Secondary": {
         "Replace": [],
         "Ignore": [
            {
               "Rule": [
                  "other"
               ],
               "Score": 0,
               "Modifier": "None",
```

```
            "Selected": false,
            "type": "Ignore",
            "u_id": "yaahbi"
          }
        ],
        "Add": []
      }
    },
    {
      "Primary": {
        "Rule": [
          "\\b carotid T \\b",
          "OR",
          "\\b carotid L \\b",
          "OR",
          "\\b carotid \"T\" \\b",
          "OR",
          "\\b carotid \"L \\b\""
        ],
        "Score": 3,
        "Selected": false,
        "type": "Primary",
        "u_id": "co00w"
      },
      "Secondary": {
        "Replace": [],
        "Ignore": [],
        "Add": []
      }
    },
    {
      "Primary": {
        "Rule": [
          "\\b with distal recanalization of the M2 \\b"
        ],
        "Score": 3,
        "Selected": true,
        "type": "Primary",
        "u_id": "p6zx3"
      },
      "Secondary": {
        "Replace": [],
        "Ignore": [],
        "Add": []
      }
    }
  ]
}
```

Supplemental Figure 2 Rules for anterior distal occlusion

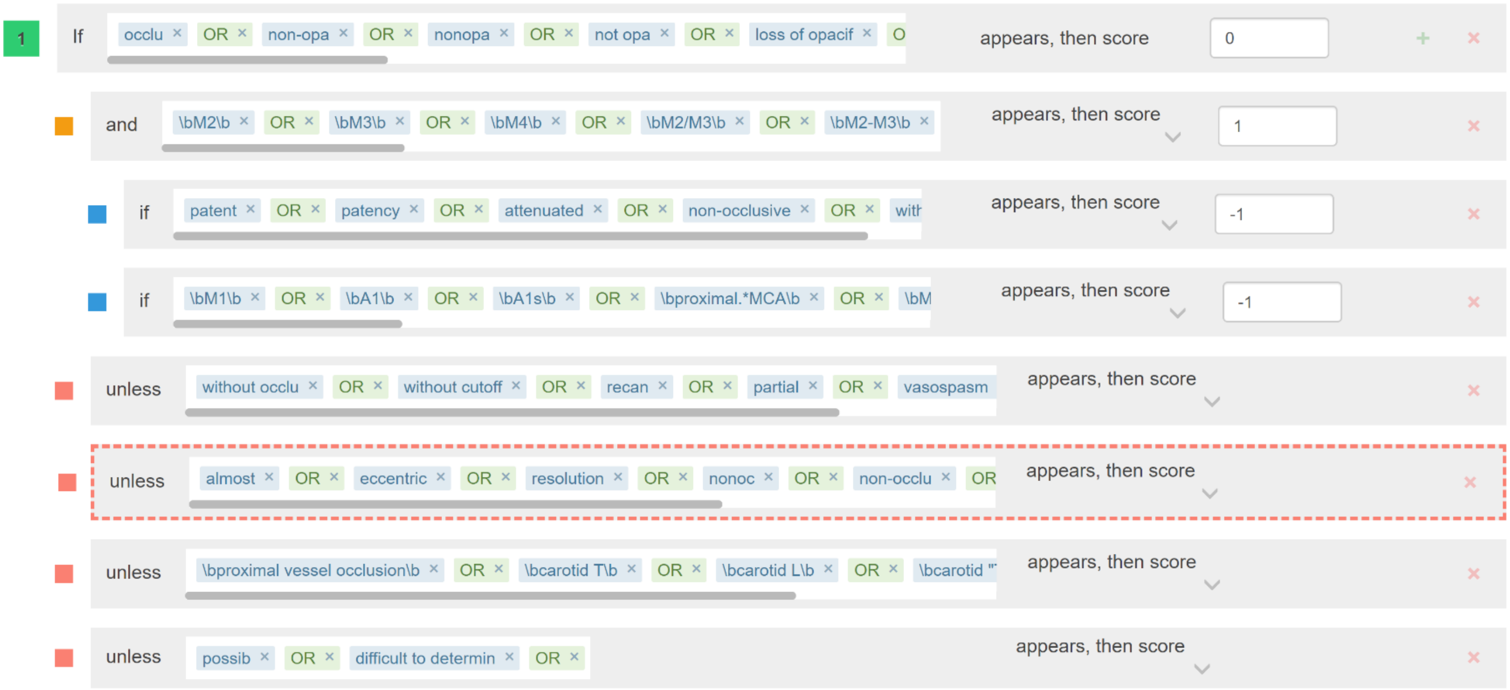

Rules for anterior distal occlusion (text format):


```
{
  "Name": "1",
  "Dirty": false,
  "Rules": [
    {
      "Primary": {
        "Rule": [
          "occlu",
          "OR",
          "non-opa",
          "OR",
          "nonopa",
          "OR",
          "not opa",
          "OR",
          "loss of opacif",
          "OR",
          "non-fill",
          "OR",
          "loss of filling",
          "OR",
          "\\babsence.*filling\\b",
          "OR",
          "abrupt termination",
          "OR",
          "cut off",
          "OR",
```

"cutoff",
"OR",
"filling defect",
"OR",
"thrombus"
],
"Score": 0,
"Selected": false,
"type": "Primary",
"u_id": "84e9x"
},
"Secondary": {
"Replace": [
{
"Rule": [
"\\bM2\\b",
"OR",
"\\bM3\\b",
"OR",
"\\bM4\\b",
"OR",
"\\bM2/M3\\b",
"OR",
"\\bM2-M3\\b",
"OR",
"\\bA2\\b",
"OR",
"\\bA3\\b",
"OR",
"\\bA4\\b",
"OR",
"\\bdistal.*MCA\\b",
"OR",
"\\b distal.*ACA\\b",
"OR",
"opercular branch",
"OR",
"insular branch",
"OR",
"callosomarginal",
"OR",
"sylvian"
],
"Score": 1,
"Modifier": "None",
"Selected": false,
"type": "Replace",
"u_id": "qhq927"
}
],
"Ignore": [
{
"Rule": [
"without occlu",
"OR",
"without cutoff",
"OR",
"recan",

```
        "OR",
        "partial",
        "OR",
        "vasospasm",
        "OR",
        "no definit"
      ],
      "Score": 0,
      "Modifier": "None",
      "Selected": false,
      "type": "Ignore",
      "u_id": "riwbu5"
    },
    {
      "Rule": [
        "almost",
        "OR",
        "eccentric",
        "OR",
        "resolution",
        "OR",
        "nonoclusive",
        "OR",
        "near",
        "OR",
        "partially"
      ],
      "Score": 0,
      "Modifier": "None",
      "Selected": false,
      "type": "Ignore",
      "u_id": "911tyh"
    },
    {
      "Rule": [
        "\\bproximal vessel occlusion\\b",
        "OR",
        "\\bcarotid T\\b",
        "OR",
        "\\bcarotid L\\b",
        "OR",
        "\\bcarotid \"T\"\\b",
        "OR",
        "\\bcarotid \"L\"\\b"
      ],
      "Score": 0,
      "Modifier": "None",
      "Selected": false,
      "type": "Ignore",
      "u_id": "sajbqx"
    },
    {
      "Rule": [
        "possib",
        "OR",
        "difficult to determin",
        "OR"
      ],
```

"Score": 0,
"Modifier": "None",
"Selected": true,
"type": "Ignore",
"u_id": "obx8kr"
}
],
"Add": [
{
"Rule": [
"patent",
"OR",
"patency",
"OR",
"attenuated",
"OR",
"non-occlusive",
"OR",
"without"
],
"Score": -1,
"Modifier": "None",
"Selected": false,
"type": "Add",
"u_id": "z24f9n"
},
{
"Rule": [
"\\bM1\\b",
"OR",
"\\bA1\\b",
"OR",
"\\bA1s\\b",
"OR",
"\\bproximal.*MCA\\b",
"OR",
"\\bMCA bifurcation\\b",
"OR",
"\\bproximal.*ACA\\b",
"OR",
"\\bICA/MCA\\b",
"OR",
"\\bMCA/ICA\\b",
"OR",
"\\bM1/M2\\b",
"OR",
"\\bM1-M2\\b",
"OR",
"petrous",
"OR",
"\\bICA\\b",
"OR",
"internal carotid artery"
],
"Score": -1,
"Modifier": "None",
"Selected": false,
"type": "Add",

```
            "u_id": "jmvp3b"
          }
        ]
      }
    }
  ]
}
```

Supplemental Figure 3 Rules for basilar occlusion

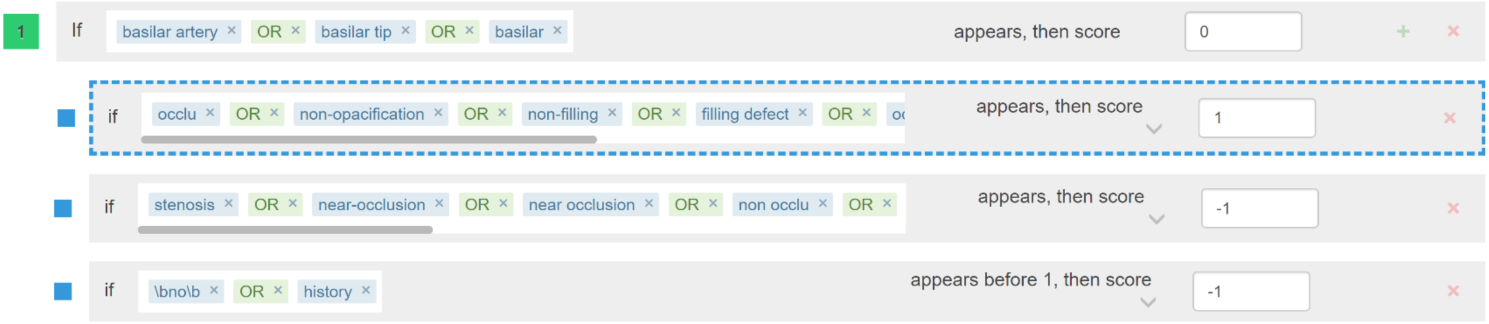

Rules for basilar occlusion (text format):


```
{
  "Name": "1",
  "Dirty": false,
  "Rules": [
    {
      "Primary": {
        "Rule": [
          "basilar artery",
          "OR",
          "basilar tip",
          "OR",
          "basilar"
        ],
        "Score": 0,
        "Selected": false,
        "type": "Primary",
        "u_id": "xs86f"
      },
      "Secondary": {
        "Replace": [],
        "Ignore": [],
        "Add": [
          {
            "Rule": [
              "occlu",
              "OR",
              "non-opacification",
              "OR",
              "non-filling",
              "OR",
              "filling defect",
              "OR",
              "occlusive thrombus",
              "OR",
              "thrombus",
              "OR",
              "thrombosis"
            ],
            "Score": 1,
            "Modifier": "None",
            "Selected": false,
            "type": "Add",
            "u_id": "ggzxch"
```

```
                },
                {
                    "Rule": [
                        "stenosis",
                        "OR",
                        "near-occlusion",
                        "OR",
                        "near occlusion",
                        "OR",
                        "non occlu",
                        "OR",
                        "without evidence of",
                        "OR",
                        "nearly",
                        "OR",
                        "almost",
                        "OR",
                        "recanaliz",
                        "OR",
                        "perfus",
                        "OR",
                        "congenital",
                        "OR",
                        "\\bcannot.*excluded\\b"
                    ],
                    "Score": -1,
                    "Modifier": "None",
                    "Selected": true,
                    "type": "Add",
                    "u_id": "2nrnig"
                },
                {
                    "Rule": [
                        "\\bno\\b",
                        "OR",
                        "history"
                    ],
                    "Score": -1,
                    "Modifier": "b",
                    "Selected": false,
                    "type": "Add",
                    "u_id": "5awu7"
                }
            ]
        }
    }
  ]
}
```

Supplemental Figure 4 Rules for presence of established ischemia

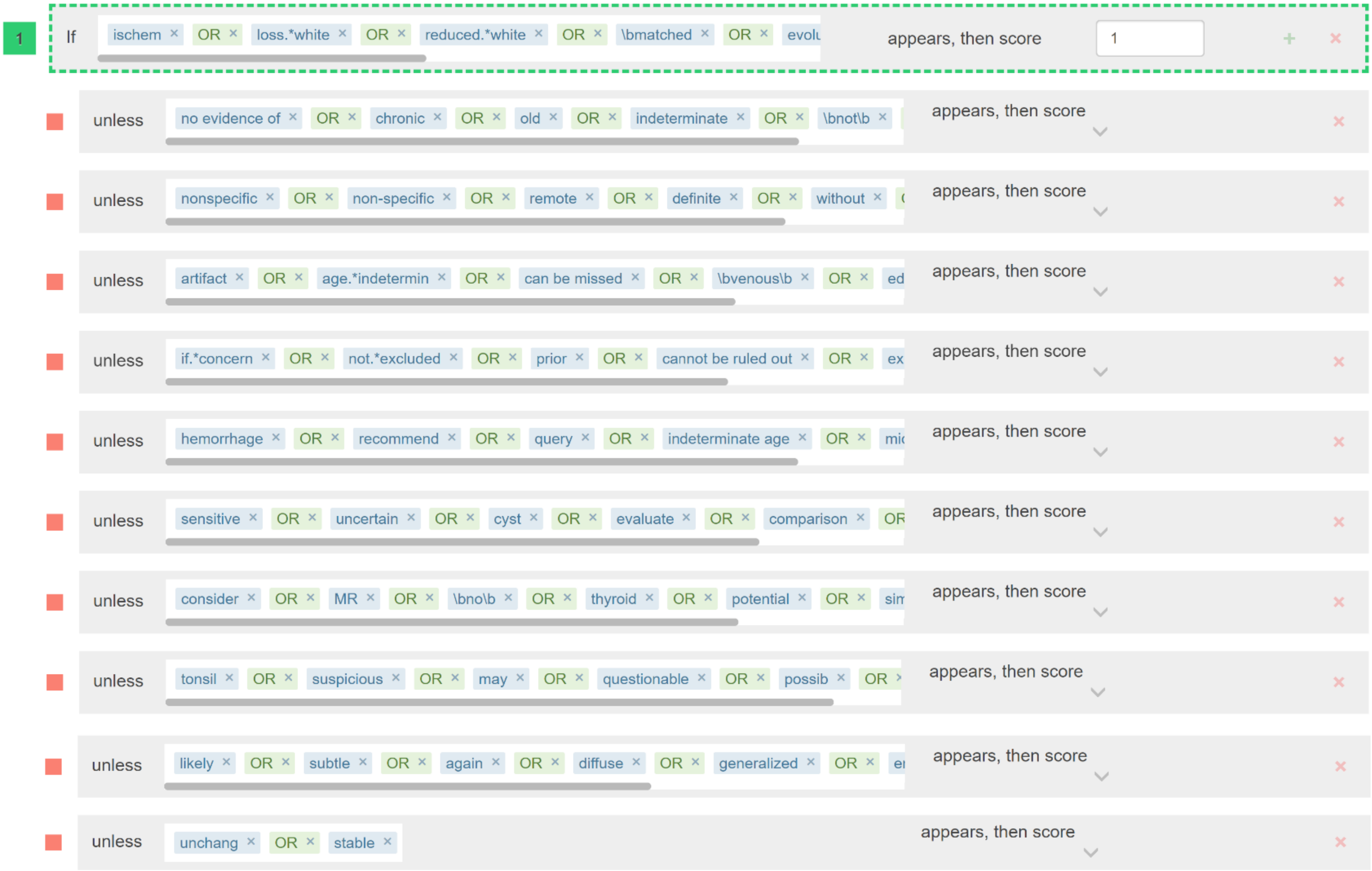

Rules for presence of established ischemia (text format):

{
  "Name": "2",
  "Dirty": false,
  "Rules": [
    {
      "Primary": {
        "Rule": [
          "ischem",
          "OR",
          "loss.*white",
          "OR",
          "reduced.*white",
          "OR",
          "\\bmatched",
          "OR",
          "evolution",
          "OR",
          "subacute",
          "OR",
          "infarct",
          "OR",
          "hypodensity",
          "OR",

"reduced atten",
"OR",
"loss.*diff"
],
"Score": 1,
"Selected": true,
"type": "Primary",
"u_id": "4jcpvo"
},
"Secondary": {
"Replace": [],
"Ignore": [
{
"Rule": [
"no evidence of",
"OR",
"chronic",
"OR",
"old",
"OR",
"indeterminate",
"OR",
"\\bnot\\b",
"OR",
"early"
],
"Score": 0,
"Modifier": "None",
"Selected": false,
"type": "Ignore",
"u_id": "a91kvh"
},
{
"Rule": [
"nonspecific",
"OR",
"non-specific",
"OR",
"remote",
"OR",
"definite",
"OR",
"without",
"OR",
"previous"
],
"Score": 0,
"Modifier": "None",
"Selected": false,
"type": "Ignore",
"u_id": "7v1qqv"
},
{
"Rule": [
"artifact",
"OR",
"age.*indetermin",
"OR",

"can be missed",
"OR",
"\\bvenous\\b",
"OR",
"edema",
"OR",
"contusion"
],
"Score": 0,
"Modifier": "None",
"Selected": false,
"type": "Ignore",
"u_id": "sz72on"
},
{
"Rule": [
"if.*concern",
"OR",
"not.*excluded",
"OR",
"prior",
"OR",
"cannot be ruled out",
"OR",
"exclude",
"OR",
"hematoma"
],
"Score": 0,
"Modifier": "None",
"Selected": false,
"type": "Ignore",
"u_id": "iizffw"
},
{
"Rule": [
"hemorrhage",
"OR",
"recommend",
"OR",
"query",
"OR",
"indeterminate age",
"OR",
"microangiopathic"
],
"Score": 0,
"Modifier": "None",
"Selected": false,
"type": "Ignore",
"u_id": "v8xhu"
},
{
"Rule": [
"sensitive",
"OR",
"uncertain",
"OR",

```
      "cyst",
      "OR",
      "evaluate",
      "OR",
      "comparison",
      "OR",
      "poorly visualized"
    ],
    "Score": 0,
    "Modifier": "None",
    "Selected": false,
    "type": "Ignore",
    "u_id": "061ov"
  },
  {
    "Rule": [
      "consider",
      "OR",
      "MR",
      "OR",
      "\\bno\\b",
      "OR",
      "thyroid",
      "OR",
      "potential",
      "OR",
      "similar",
      "OR",
      "mismatch"
    ],
    "Score": 0,
    "Modifier": "None",
    "Selected": false,
    "type": "Ignore",
    "u_id": "ynnnks"
  },
  {
    "Rule": [
      "tonsil",
      "OR",
      "suspicious",
      "OR",
      "may",
      "OR",
      "questionable",
      "OR",
      "possib",
      "OR",
      "can"
    ],
    "Score": 0,
    "Modifier": "None",
    "Selected": false,
    "type": "Ignore",
    "u_id": "dh1gy"
  },
  {
    "Rule": [
```

```
                "likely",
                "OR",
                "subtle",
                "OR",
                "again",
                "OR",
                "diffuse",
                "OR",
                "generalized",
                "OR",
                "encephalom",
                "OR",
                "since",
                "OR",
                "myocard"
              ],
              "Score": 0,
              "Modifier": "None",
              "Selected": false,
              "type": "Ignore",
              "u_id": "er01nq"
            },
            {
              "Rule": [
                "unchang",
                "OR",
                "stable"
              ],
              "Score": 0,
              "Modifier": "None",
              "Selected": false,
              "type": "Ignore",
              "u_id": "a4xj2f"
            }
          ],
          "Add": []
        }
      }
    ]
  }
```

Supplemental Figure 5 Rules for presence of any hemorrhage

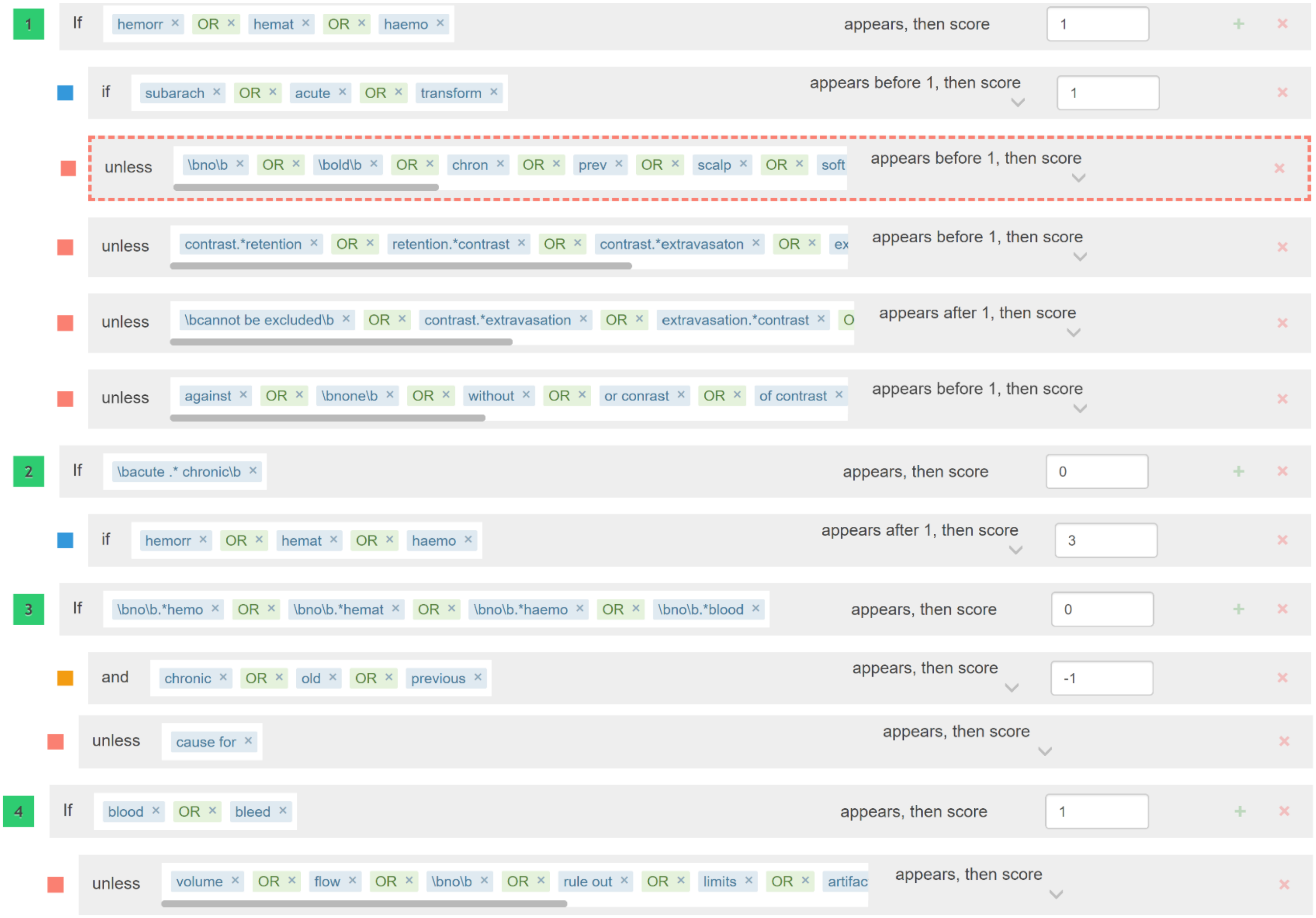

Rules for presence of any hemorrhage (text format):

{
  "Name": "1",
  "Dirty": false,
  "Rules": [
    {
      "Primary": {
        "Rule": [
          "hemorr",
          "OR",
          "hemat",
          "OR",
          "haemo"
        ],
        "Score": 1,
        "Selected": false,
        "type": "Primary",
        "u_id": "k1p17i"
      },
      "Secondary": {

```
"Replace": [],
"Ignore": [
  {
    "Rule": [
      "\\bno\\b",
      "OR",
      "\\bold\\b",
      "OR",
      "chron",
      "OR",
      "prev",
      "OR",
      "scalp",
      "OR",
      "soft tissue",
      "OR",
      "intramuscular",
      "OR",
      "extravascular",
      "OR",
      "\\bnot\\b",
      "OR",
      "without",
      "OR",
      "intramural",
      "OR",
      "\\bno.*change\\b"
    ],
    "Score": 0,
    "Modifier": "b",
    "Selected": true,
    "type": "Ignore",
    "u_id": "4nzvq8"
  },
  {
    "Rule": [
      "contrast.*retention",
      "OR",
      "retention.*contrast",
      "OR",
      "contrast.*extravasaton",
      "OR",
      "extravasation.*contrast",
      "OR",
      "suspected"
    ],
    "Score": 0,
    "Modifier": "b",
    "Selected": false,
    "type": "Ignore",
    "u_id": "ny8hnk"
  },
  {
    "Rule": [
      "\\bcannot be excluded\\b",
      "OR",
      "contrast.*extravasation",
      "OR",
```

```
            "extravasation.*contrast",
            "OR",
            "or contrast",
            "OR",
            "of contrast",
            "OR",
            "\\bcannot be ruled out\\b",
            "OR",
            "not seen"
        ],
        "Score": 0,
        "Modifier": "a",
        "Selected": false,
        "type": "Ignore",
        "u_id": "im76r4"
    },
    {
        "Rule": [
            "against",
            "OR",
            "\\bnone\\b",
            "OR",
            "without",
            "OR",
            "or conrast",
            "OR",
            "of contrast",
            "OR",
            "may",
            "OR",
            "limits.*sensitivity",
            "OR",
            "last study",
            "OR",
            "\\blimits\\b",
            "OR",
            "rather than"
        ],
        "Score": 0,
        "Modifier": "b",
        "Selected": false,
        "type": "Ignore",
        "u_id": "m0fnjc"
    }
],
"Add": [
    {
        "Rule": [
            "subarach",
            "OR",
            "acute",
            "OR",
            "transform"
        ],
        "Score": 1,
        "Modifier": "b",
        "Selected": false,
        "type": "Add",
```

```
                "u_id": "b4866u"
            }
        ]
    }
},
{
    "Primary": {
        "Rule": [
            "\\bacute .* chronic\\b"
        ],
        "Score": 0,
        "Selected": false,
        "type": "Primary",
        "u_id": "gvnxj"
    },
    "Secondary": {
        "Replace": [],
        "Ignore": [],
        "Add": [
            {
                "Rule": [
                    "hemorr",
                    "OR",
                    "hemat",
                    "OR",
                    "haemo"
                ],
                "Score": 3,
                "Modifier": "a",
                "Selected": false,
                "type": "Add",
                "u_id": "c63b7z"
            }
        ]
    }
},
{
    "Primary": {
        "Rule": [
            "\\bno\\b.*hemo",
            "OR",
            "\\bno\\b.*hemat",
            "OR",
            "\\bno\\b.*haemo",
            "OR",
            "\\bno\\b.*blood"
        ],
        "Score": 0,
        "Selected": false,
        "type": "Primary",
        "u_id": "d6r8af"
    },
    "Secondary": {
        "Replace": [
            {
                "Rule": [
                    "chronic",
                    "OR",
```

"old",
"OR",
"previous"
],
"Score": -1,
"Modifier": "None",
"Selected": false,
"type": "Replace",
"u_id": "44l2f8"
}
],
"Ignore": [
{
"Rule": [
"cause for"
],
"Score": 0,
"Modifier": "None",
"Selected": false,
"type": "Ignore",
"u_id": "nle6pa"
}
],
"Add": []
}
},
{
"Primary": {
"Rule": [
"blood",
"OR",
"bleed"
],
"Score": 1,
"Selected": false,
"type": "Primary",
"u_id": "mtzbqf"
},
"Secondary": {
"Replace": [],
"Ignore": [
{
"Rule": [
"volume",
"OR",
"flow",
"OR",
"\\bno\\b",
"OR",
"rule out",
"OR",
"limits",
"OR",
"artifact",
"OR",
"cannot be excluded",
"OR",
"pressure",

```
                "OR",
                "sugar"
              ],
              "Score": 0,
              "Modifier": "None",
              "Selected": false,
              "type": "Ignore",
              "u_id": "b3bvcv"
            }
          ],
          "Add": []
        }
      }
    ]
}
```

Supplemental Figure 6 Rules for Alberta stroke program early CT score (binary)

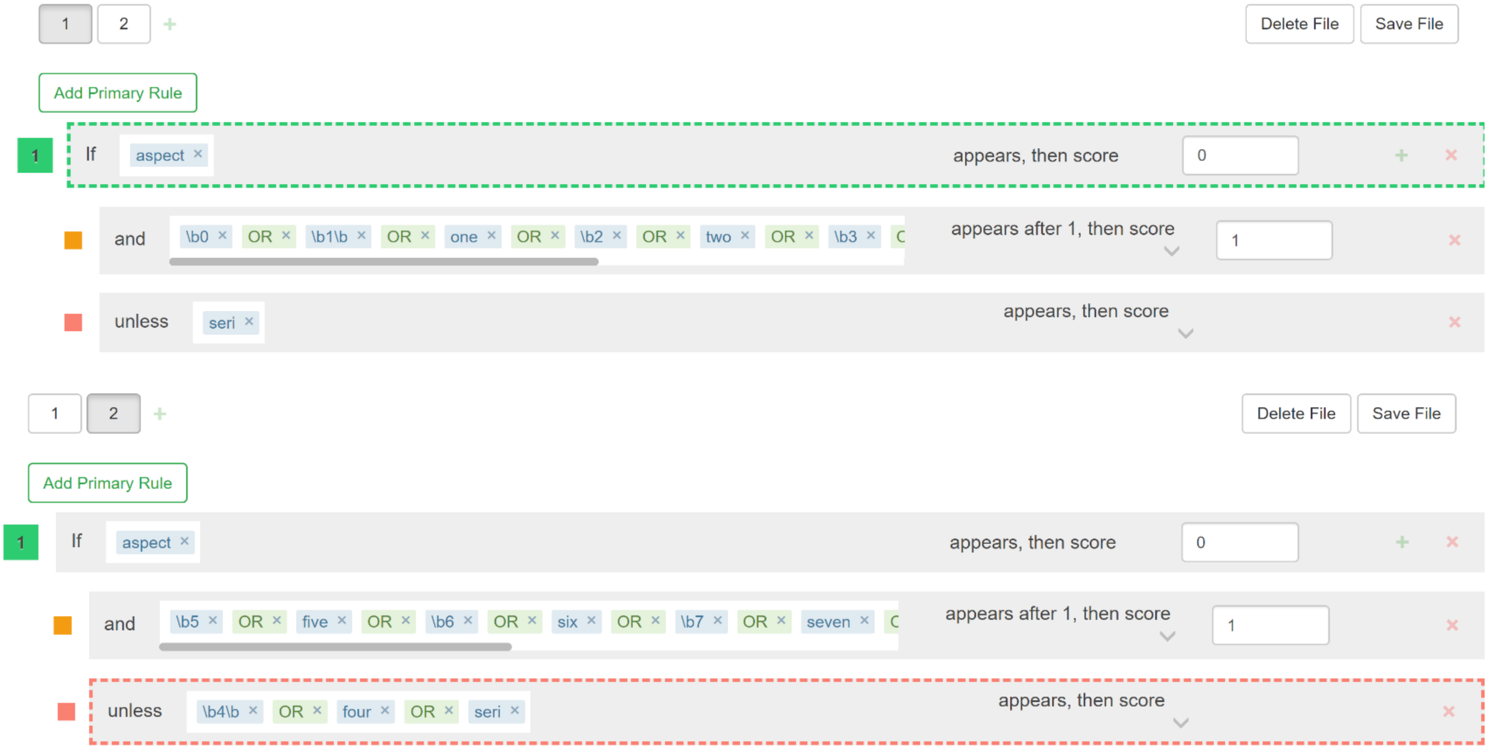

Rules for Alberta stroke program early CT score (text format):


```
{
  "Name": "1",
  "Dirty": false,
  "Rules": [
    {
      "Primary": {
        "Rule": [
          "aspect"
        ],
        "Score": 0,
        "Selected": false,
        "type": "Primary",
        "u_id": "676aj"
      },
      "Secondary": {
        "Replace": [
          {
            "Rule": [
              "\\b0\\b",
              "OR",
              "zero",
              "OR",
              "\\b1\\b",
              "OR",
              "\\bone",
              "OR",
```

```
                    "\\b2",
                    "OR",
                    "\\btwo",
                    "OR",
                    "\\b3",
                    "OR",
                    "\\bthree",
                    "OR",
                    "\\b4",
                    "OR",
                    "\\bfour",
                    "OR",
                    "\\b4-\\b"
                ],
                "Score": 1,
                "Modifier": "a",
                "Selected": true,
                "type": "Replace",
                "u_id": "gbfu7c"
            }
        ],
        "Ignore": [
            {
                "Rule": [
                    "seri",
                    "OR",
                    "image"
                ],
                "Score": 0,
                "Modifier": "None",
                "Selected": false,
                "type": "Ignore",
                "u_id": "vzllm"
            }
        ],
        "Add": []
    }
  }
 ]
}

{
 "Name": "2",
 "Dirty": false,
 "Rules": [
  {
   "Primary": {
    "Rule": [
     "aspect"
    ],
    "Score": 0,
    "Selected": false,
    "type": "Primary",
    "u_id": "m0oex7"
   },
   "Secondary": {
    "Replace": [
     {
```

```
                "Rule": [
                    "\\b5",
                    "OR",
                    "five",
                    "OR",
                    "\\b6",
                    "OR",
                    "six",
                    "OR",
                    "\\b7",
                    "OR",
                    "seven",
                    "OR",
                    "\\b8",
                    "OR",
                    "eight",
                    "OR",
                    "\\b9",
                    "OR",
                    "nine",
                    "OR",
                    "\\b10\\b",
                    "OR",
                    "\\bten\\b"
                ],
                "Score": 1,
                "Modifier": "a",
                "Selected": false,
                "type": "Replace",
                "u_id": "n1uc7a"
            }
        ],
        "Ignore": [
            {
                "Rule": [
                    "\\b4\\b",
                    "OR",
                    "four",
                    "OR",
                    "seri"
                ],
                "Score": 0,
                "Modifier": "None",
                "Selected": true,
                "type": "Ignore",
                "u_id": "bxj4si"
            }
        ],
        "Add": []
    }
  }
 ]
}
```

Supplemental Figure 7 Rules for collateral status

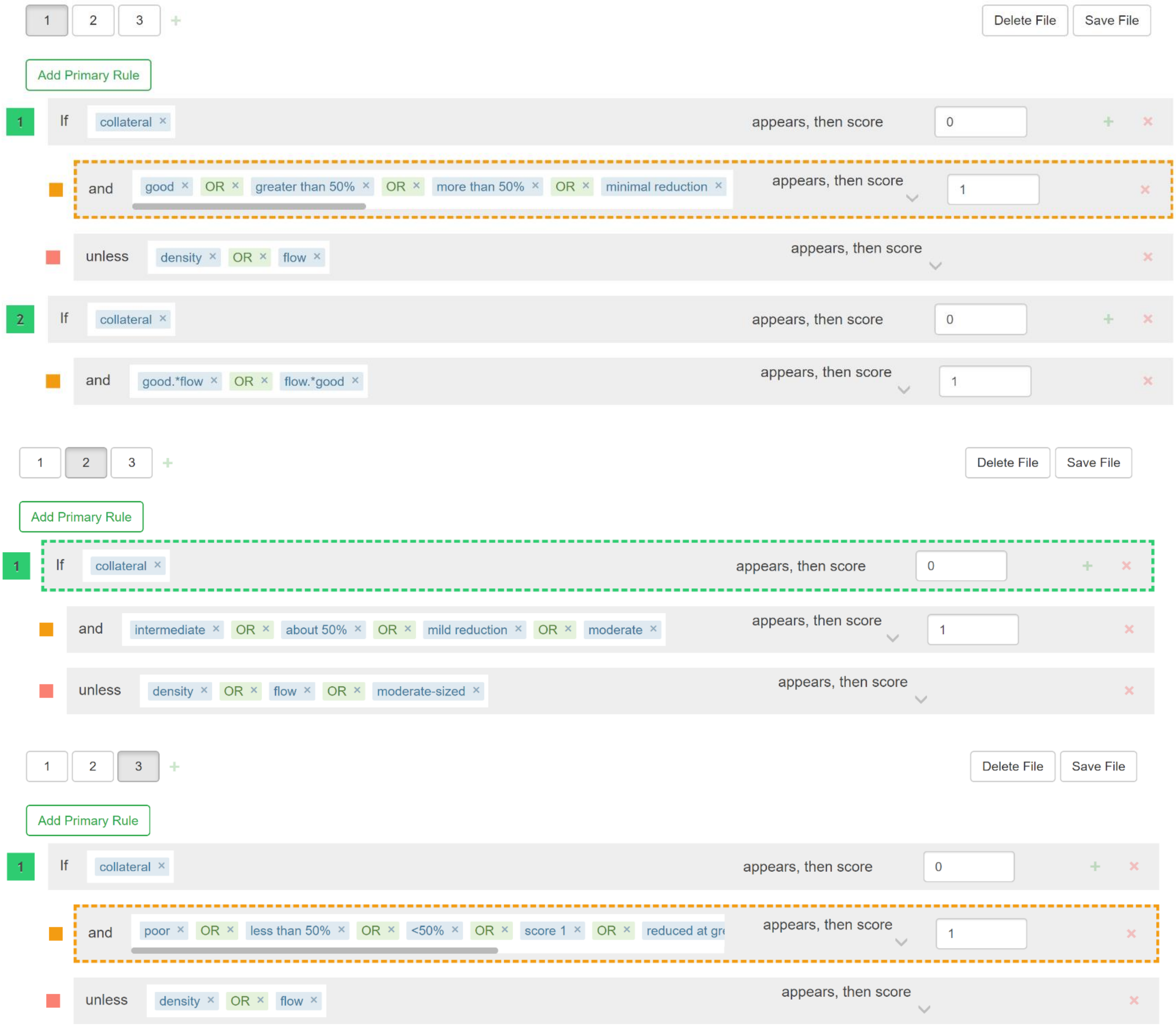

Rules for collateral status (text format):

{
    "Name": "1",
    "Dirty": false,
    "Rules": [
        {
            "Primary": {
                "Rule": [
                    "collateral"

],
"Score": 0,
"Selected": false,
"type": "Primary",
"u_id": "jgmjtg"
},
"Secondary": {
"Replace": [
{
"Rule": [
"good",
"OR",
"greater than 50%",
"OR",
"more than 50%",
"OR",
"minimal reduction",
"OR",
"filling >50%",
"OR",
">50%",
"OR",
"adequate",
"OR",
"preserved",
"OR",
"excellent",
"OR",
"exceed 50%",
"OR",
"score 2"
],
"Score": 1,
"Modifier": "None",
"Selected": true,
"type": "Replace",
"u_id": "k26tk"
}
],
"Ignore": [
{
"Rule": [
"density",
"OR",
"flow"
],
"Score": 0,
"Modifier": "None",
"Selected": false,
"type": "Ignore",
"u_id": "6nvxd"
}
],
"Add": []
}
},
{
"Primary": {

```
"Rule": [
   "collateral"
],
"Score": 0,
"Selected": false,
"type": "Primary",
"u_id": "r8u91j"
},
"Secondary": {
   "Replace": [
      {
         "Rule": [
            "good.*flow",
            "OR",
            "flow.*good"
         ],
         "Score": 1,
         "Modifier": "None",
         "Selected": false,
         "type": "Replace",
         "u_id": "40fek9"
      }
   ],
   "Ignore": [],
   "Add": []
}
}
]
}

{
   "Name": "2",
   "Dirty": false,
   "Rules": [
      {
         "Primary": {
            "Rule": [
               "collateral"
            ],
            "Score": 0,
            "Selected": true,
            "type": "Primary",
            "u_id": "8n8bq"
         },
         "Secondary": {
            "Replace": [
               {
                  "Rule": [
                     "intermediate",
                     "OR",
                     "about 50%",
                     "OR",
                     "mild reduction",
                     "OR",
                     "moderate"
                  ],
                  "Score": 1,
                  "Modifier": "None",
```

```
          "Selected": false,
          "type": "Replace",
          "u_id": "od71pt"
        }
      ],
      "Ignore": [
        {
          "Rule": [
            "density",
            "OR",
            "flow",
            "OR",
            "moderate-sized"
          ],
          "Score": 0,
          "Modifier": "None",
          "Selected": false,
          "type": "Ignore",
          "u_id": "qihd6i"
        }
      ],
      "Add": []
    }
  }
 ]
}

{
 "Name": "3",
 "Dirty": false,
 "Rules": [
  {
    "Primary": {
      "Rule": [
        "collateral"
      ],
      "Score": 0,
      "Selected": false,
      "type": "Primary",
      "u_id": "jw2wsm"
    },
    "Secondary": {
      "Replace": [
        {
          "Rule": [
            "poor",
            "OR",
            "less than 50%",
            "OR",
            "<50%",
            "OR",
            "score 1",
            "OR",
            "reduced at greater than 50%",
            "OR",
            "\\bno\\b",
            "OR",
            "slight filling"
```

```
                    ],
                    "Score": 1,
                    "Modifier": "None",
                    "Selected": true,
                    "type": "Replace",
                    "u_id": "rm7n9m"
                }
            ],
            "Ignore": [
                {
                    "Rule": [
                        "density",
                        "OR",
                        "flow"
                    ],
                    "Score": 0,
                    "Modifier": "None",
                    "Selected": false,
                    "type": "Ignore",
                    "u_id": "peva1"
                }
            ],
            "Add": []
        }
    }
  ]
}
```
